# Synthesis and Evaluation of a ZnO-Chitosan Adduct for Safe and Sustainable Enhanced Ultra-Violet (UV) Sunscreens Protection

**DOI:** 10.3390/molecules29215204

**Published:** 2024-11-03

**Authors:** Mattia Battistin, Alessandro Bonetto, Francesco Nicoli, Elena Torreggiani, Andrea Brunetta, Elena Cesa, Stefano Manfredini, Anna Baldisserotto, Silvia Vertuani

**Affiliations:** 1 Kalis S.r.l., 31040 Onigo di Pederobba (TV), Italy; mattia.battistin@kalis.it (M.B.); andrea.brunetta@kalis.it (A.B.); 2 DAIS—Department of Environmental Sciences, Informatics and Statistics, University Ca’ Foscari of Venice, 30172 Venice, Italy; alessandro.bonetto@unive.it; 3 Department of Chemical, Pharmaceutical and Agricultural Sciences, University of Ferrara, 44100 Ferrara, Italy; francesco.nicoli@unife.it (F.N.); elena.torreggiani@unife.it (E.T.); 4 Department of Life Sciences and Biotechnology, University of Ferrara, 44100 Ferrara, Italy; elena.cesa@unife.it (E.C.); bldnna@unife.it (A.B.); vrs@unife.it (S.V.); 5 Ambrosialab S.r.l., 44121 Ferrara, Italy

**Keywords:** cosmeceutical, chitosan, zinc oxide, free radicals, UV sunscreen filters, UV booster activity

## Abstract

Chitosan (Ch), a natural polysaccharide, is known for its biocompatibility, biodegradability, and various beneficial properties, including antioxidant and antibacterial activities. The objective of this study is to investigate the functionalization of zinc oxide (ZnO) with chitosan to develop a novel ZnO@Ch adduct for use in cosmetic formulations, specifically as a sun protection agent. The functionalization was achieved through ionotropic gelation, which enhanced the stability and reduced the photocatalytic activity of ZnO, thereby improving its safety profile for skin applications. FTIR spectroscopy confirmed the successful functionalization, while TGA and DSC characterized the thermal properties and stability. The Zeta potential and particle size analyses demonstrated improved stability of ZnO@Ch across various pH levels compared to uncoated ZnO. The structure of the obtained adduct was also confirmed by SEM analysis. The ZnO@Ch adduct exhibited enhanced stability at neutral and slightly alkaline pH values, reduced photocatalytic activity compared to pure ZnO, and had lower cytotoxicity in 3T3 cells compared to pure ZnO, particularly at higher concentrations. The ZnO@Ch adduct provided a higher Sun Protection Factor (SPF) and UVA Protection Factor (UVA-PF) than pure ZnO, indicating enhanced UV protection. The adduct’s ability to provide higher SPF at lower ZnO concentrations offers economic and environmental benefits, aligning with sustainable product design principles. Future studies will focus on optimizing the formulation and testing the efficacy and safety at higher concentrations to fully realize its potential as a natural, eco-friendly sunscreen ingredient.

## 1. Introduction

Chitosan (Ch) is a natural polysaccharide derived from deacetylated chitin. It is a linear copolymer of 2-acetamido-2-deoxy-β-d-glucopyranose and 2-amino-2-deoxy-β-d-glucopyranose. Ch films have positively charged amino groups that interact with negatively charged components of the membrane, promoting cell adhesion and reducing the loss of intracellular material. Charged groups of Ch allow for controlled mechanical release to occur [1]. Notable properties of Ch include biocompatibility, biodegradability, hydrophilicity, good toxicity profile, organoleptic properties, high bioavailability, ease of molecular modification, and favorable water permselectivity [2]. Ch possesses exceptional properties, such as chemical resistance, the ability to form various structures, and a strong affinity for metals, proteins, and dyes. Ch is broken down in the human body into safe compounds that are easily absorbed. Its film-forming and skin-protective properties are widely utilized in dietetics, dermatology, and cosmetology [3]. Ch moisturizes the skin, even in low humidity conditions, and protects it from external agents [4]. Ch has gained significant attention due to its potent antioxidant and antibacterial properties [5,6]. Chitosan derivatives, which have an amine group and two hydroxyl groups, display enhanced antioxidant activity by neutralizing free radicals and reactive oxygen species. Ismailovi et al. [7], indicate that a high molecular weight chitosan was used to prepare an optimal gel formulation with significant antioxidant activity, suggesting that a higher molecular weight does not necessarily impede antioxidant performance [8]. Furthermore, modifications to chitosan, such as the addition of functional groups or nanoparticles, can enhance its antioxidant activity, irrespective of the molecular weight [9]. In recent years, the term “booster” has gained popularity in the cosmetic industry, referring to ingredients that enhance or sustain the effectiveness of other ingredients in a formulation. Booster ingredients, such as small molecules, polymers, and particles, work in conjunction with other ingredients to increase the effectiveness of a product [10]. For instance, booster molecules in sunscreen products enhance the SPF of the formulation. The use of booster ingredients has been shown to improve the effectiveness of sunscreen products in protecting against UV radiation.

Boosters are commonly designed to interact with UV filters or the rheological properties of a formulation [11].

This also includes the use of antioxidant substances such as chitosan, which can improve UV filtering capacity, reduce oxidative stress, and protect DNA from free radical generation, and thus reduce skin ageing [12]. In recent years, there has been a growing demand for natural, sustainable, and biodegradable sunscreens. To tackle this problem, environmentally friendly alternatives are needed, such as natural polymers (e.g., chitosan) [13]. Mineral filters, such as zinc oxide (ZnO) functionalized with chitosan, offer a promising solution.

The pharmaceutical and cosmetic industries, particularly the sunscreen sector, have witnessed a surge in demand for natural, eco-friendly, and biodegradable products. This trend has arisen due to the growing awareness of the detrimental effects of traditional sunscreens on aquatic ecosystems [14,15,16]. Researchers are developing solutions that are both effective and eco-friendly. Studies have shown that molecules used in sunscreens have a detrimental impact on marine life, making this a pressing issue [17]. However, the advantages of sunscreen cannot be overlooked. Scientists are currently investigating potential alternatives to conventional sunscreens, which are often prone to instability and photoreactivity [18]. Moreover, there is a growing trend towards incorporating natural ingredients with anti-aging properties into sunscreen formulations [19,20]. This study describes an environmentally friendly approach developed in partnership with the European projects SbD4Nano and SUNRISE, and the University of Ferrara’s academic spin-off company called Ambrosialab.

The research explores the functionalization of zinc oxide with chitosan, focusing on the following two main aspects: synthesis and formulation of the adduct, and instrumental evaluation of its properties. To create the ZnO@Ch adduct, an ionotropic gelation technique using pentasodium triphosphate (TPP) was employed. This method enables the production of polymeric systems that are capable of encapsulating various substances and finding applications in food, pharmaceuticals, and cosmetics.

## 2. Results

### 2.1. Synthesis of Coated ZnO

**Figure 1:**
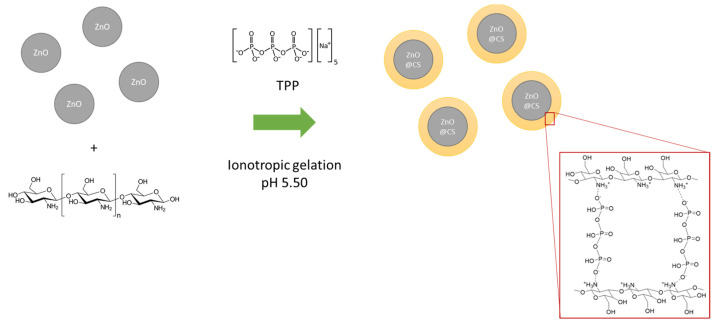
Ionotropic gelation with chitosan for ZnO.

The functionalization process for ZnO molecules involves a reaction that imparts specific reactivity, thereby altering its biological properties and enabling it to be utilized for a variety of purposes. This functionalization process was achieved through ionotropic gelation (Figure 1), as described in the Materials and Methods section (Section 4.2).

### 2.2. Fourier Transform Infrared (FTIR) Spectroscopy

**Figure 2:**
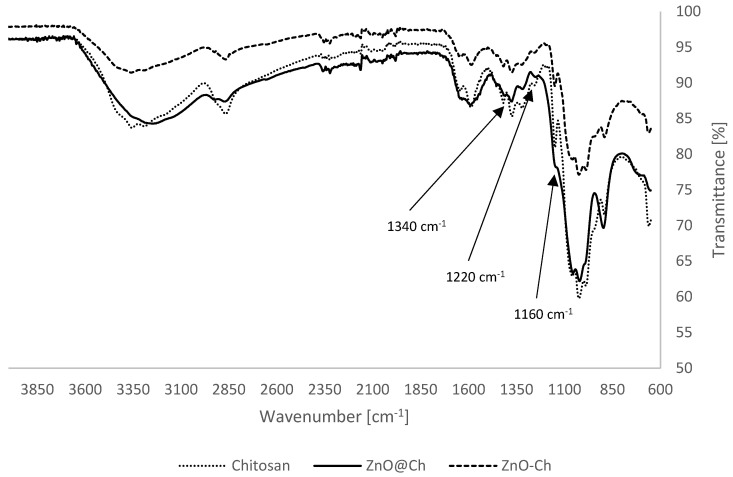
FT-IR spectra profiles.

FT-IR analyses provided the following spectra, enabling a comparison between the synthesized adduct (ZnO@Ch), pure chitosan, and a mixture of chitosan and zinc oxide (ZnO-Ch). This comparison allows for a more immediate and timely qualitative analysis of the actual functionalization (Figure 2).

The spectra indicate an effective discontinuity between the pure chitosan and mixture in relation to the adduct. Notably, a decrease in the peak at 1160 cm^−1^, attributed to the stretching of alcoholic C-O, is evident. Additionally, a decrease at 1340 cm^−1^ and 1220 cm^−1^, related to the stretching of amine C-N, was also observed. These changes are consistent with the functionalization of ZnO.

### 2.3. Thermogravimetric Analysis and Differential Scanning Calorimetry

The ZnO@Ch adduct underwent Thermogravimetric Analysis and Differential Scanning Calorimetry.

The Thermogravimetric Analysis revealed that the primary weight loss occurred between 100–120 °C, which was attributed to moisture evaporation. At approximately 450 °C, the weight decreased to about 55%. Differential Scanning Calorimetry demonstrated an enthalpy peak at 250 °C, and a less pronounced peak around 350 °C. The presence of these two peaks might suggest the formation of two different chitosan species, although the second peak was considered insignificant. This was confirmed by the fact that the combustion of different hypothetical species did not influence the degradation profile of the binder, as made evident by the thermogravimetric curve [21]. The data obtained from the graphs are listed in (Table 1).

The functionalization values were relatively high, exceeding 40% of the total particle weight. Such values can be advantageous during the polymerization process around the ZnO core, as the process operated by chitosan differs from a simple coating.

### 2.4. Colloidal Characthterization

To compare the behavior of ZnO and ZnO@Ch in liquid matrices, the hydrodynamic diameter (particle size) and the surface charge (Z-potential) have been measured at different pHs. Indeed, the colloidal stability has been evaluated by studying the sedimentation rate by means of an analytical centrifuge.

**Figure 3:**
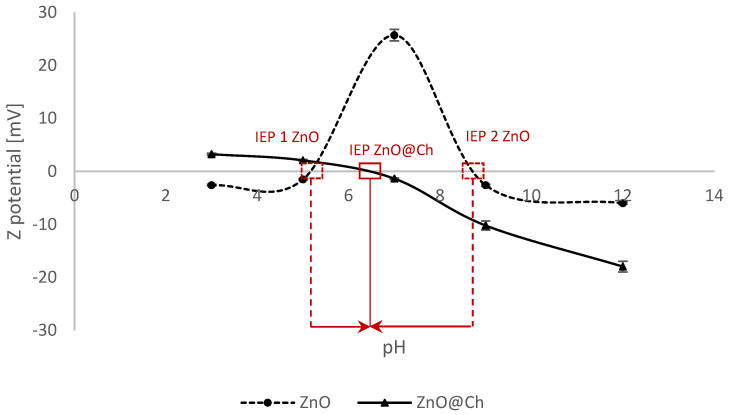
Z-potential trend for ZnO and ZnO@Ch. The isoelectric points (IEP) are highlighted. The shift to neutral pH value for the coated ZnO is evident.

Table 2 and Figure 3 display the Z-potential values of ZnO and ZnO@Ch at various pH levels.

As shown in Table 2, the Z-potential of ZnO decreases with decreasing pH, consistent with previous studies. Additionally, functionalization shifts the isoelectric point toward values closer to neutrality. In contrast, the Z-potential of ZnO@Ch remains stable across the entire pH range. While the functionalization causes a shift in the isoelectric point, it also plays a critical role in preventing the collapse of the potential under acidic conditions compared to pure ZnO. This phenomenon, which is well-documented for ZnO, is attributed to its increased solubility at extreme pH levels, leading to reduced filtering activity.

**Figure 4:**
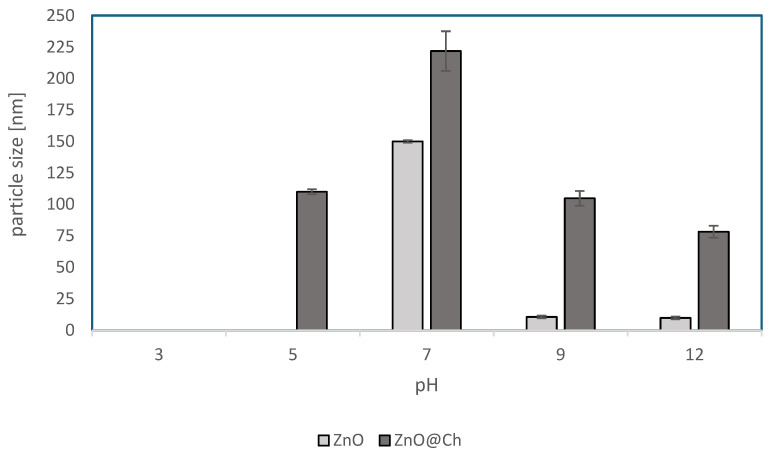
Trend of particle size changing with pH.

Table 3 and Figure 4 present the particle sizes of ZnO and ZnO@Ch at different pH values. The table shows that the particle size of ZnO decreases with decreasing pH, whereas the particle size of ZnO@Ch remains relatively constant across the pH range. At low pH values the particle size is undetectable, especially for the uncoated ZnO. This is because the low pH increases the solubility of ZnO.

These results are also confirmed by Figure 4, which shows that the particle size of ZnO decreases with an increase in the pH, whereas the particle size of ZnO@Ch remains relatively constant throughout the pH range, showing conservation of particle degradation.

It is important to note that the values obtained through the ELS technique are influenced by pH due to the solubilization of ZnO in acidic environments. Particle size measurements as a function of pH corroborate the Z-potential findings. Although particle size varies with pH for both ZnO and ZnO@Ch, this variability is significantly reduced in the coated ZnO. This confirms that the coating provides protection against ZnO solubilization, stabilizing the particles and preserving the product’s effectiveness throughout its shelf life without fluctuations in SPF.

**Figure 5:**
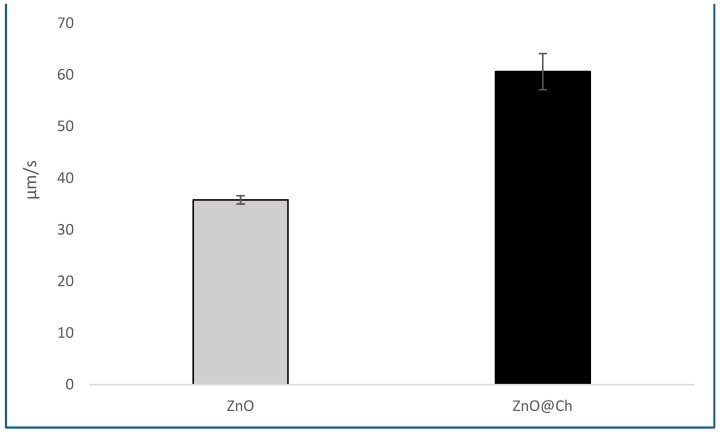
Graphical representation of sedimentation rate for coated and uncoated ZnO.

The data also showed an increase in particle size at pH 7, supported by the Z-potential, which indicates an increase in the particle diameter near the isoelectric point. This increase in particle size is likely due to coalescence at this pH value. Regarding sedimentation velocity, the sedimentation rate for ZnO was 35.78 µm/s with a deviation of 0.8, while for ZnO@Ch, the sedimentation rate was 60.62 µm/s with a deviation of 3.5. These results are presented in Table 4 and Figure 5.

Functionalization of ZnO with chitosan resulted in a significant increase in the sedimentation rate, which can be attributed to the formation of complex and polymeric species that increase particle size and decrease the stability of the suspension.

### 2.5. Surface Characterization: SEM-EDS and TEM

Chemical composition and surface particle morphologies were investigated by scanning electron microscopy (SEM), coupled with a detector for energy-dispersive X-ray spectroscopy (EDS). In addition, high resolution SEM and TEM images have been acquired.

**Figure 6:**
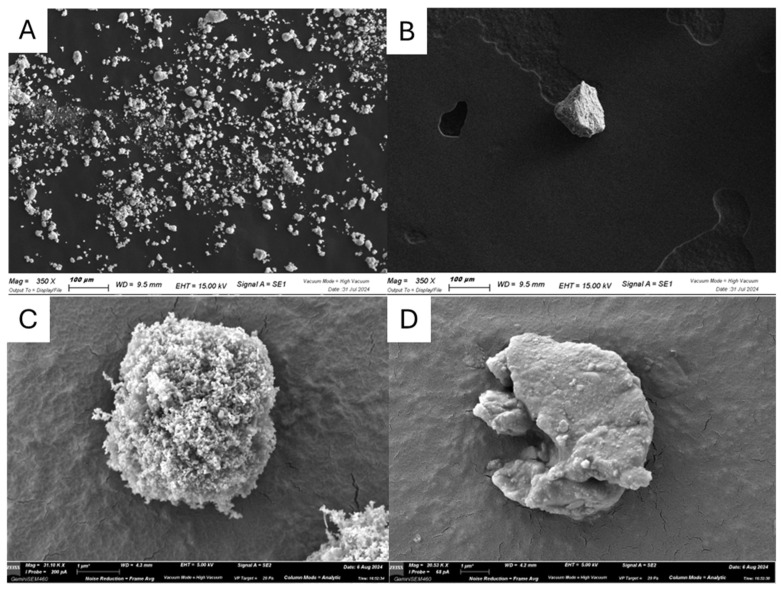
SEM image of pristine ZnO (**A**) and of functionalized ZnO@Ch (**B**); HR-SEM image of pristine ZnO (**C**) and of functionalized ZnO@Ch (**D**).

**Figure 7:**
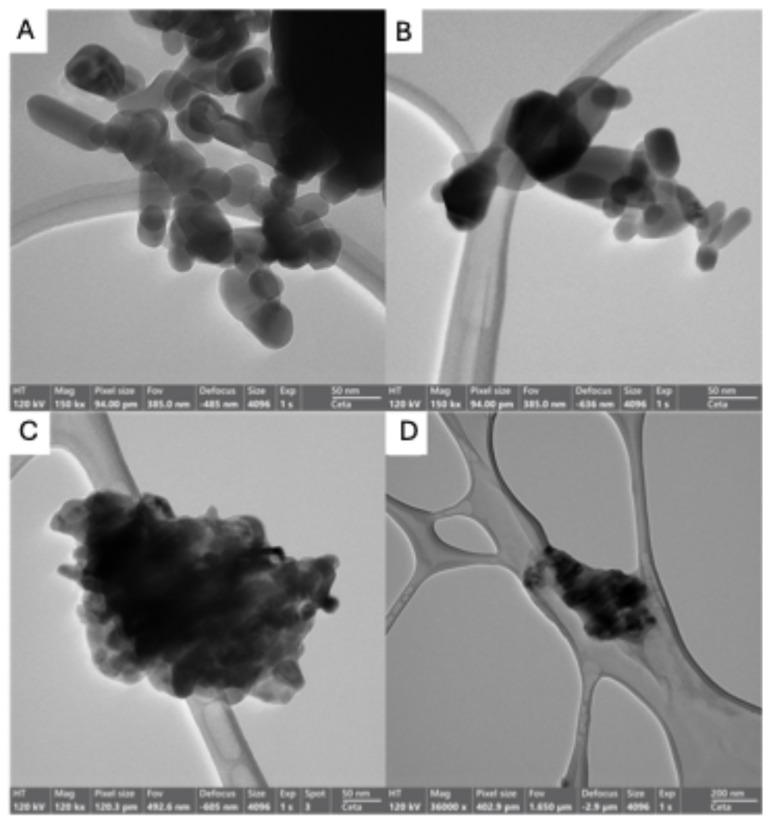
TEM images of pristine ZnO (**A**,**B**) and functionalized ZnO@Ch (**C**,**D**).

In panels A and C of Figure 6, the characteristic structure of the ZnO is shown, and it differs from the ZnO@Ch agglomerates (panels B and D), in which the matrix of chitosan embeds the ZnO particles. According to the SEM images reported in Figure 6, the TEM images reported in Figure 7 for ZnO (panels A and B) and ZnO@Ch (panels C and D) confirm the functionalization made by ionotopic gelation. In fact, in the images of the pristine material (panels A and B), the ZnO particles are clearly visible and distinguishable, while in the images of the modified ZnO@Ch (panels C and D), the agglomerates lose their resolution due to both the presence of an organic ligand (chitosan matrix) and the increase in the agglomerate dimension.

**Figure 8:**
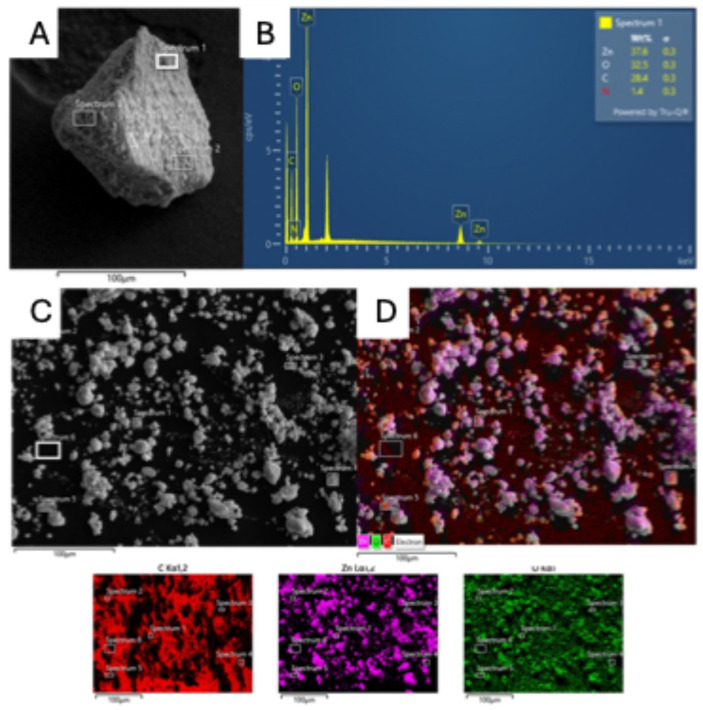
SEM-EDS map analysis of functionalized ZnO@Ch (**A**,**B**) and pristine ZnO (**C**,**D**).

A SEM-EDS analysis was performed to confirm the dual nature of the material (Figure 8).

From map analysis, it is possible to confirm the interaction that occurred; as a matter of fact, all atoms characteristic of both ZnO (Zn and O) and chitosan (N and C) coexist in the functionalized ZnO particle. The presence of N and C is precisely related to agglomeration with chitosan, as confirmed by the absence of the two in the panels M and N, which depict the pristine ZnO. It should be noted that the presence of C in panel L is only due to the carbon tape support, as the red spots are only visible in the background.

### 2.6. Photodegradation

The effective photodegradation of Acid Blue 9 [22] was determined using a calibration line, and the results are presented in Table 5, which depicts the degradation of the dye after 30 min of exposure. The amount of adduct used was measured to ensure the same amount of ZnO was available for photocatalysis. The degradation values for ZnO and ZnO@Ch are summarized in Table 5, which shows a decrease in photocatalytic activity following the functionalization of ZnO with chitosan. As depicted in the histogram presented in Figure 5, the protection induced by the coating is evident

### 2.7. Neutral Red Assay Cytotoxicity Assay

**Figure 9:**
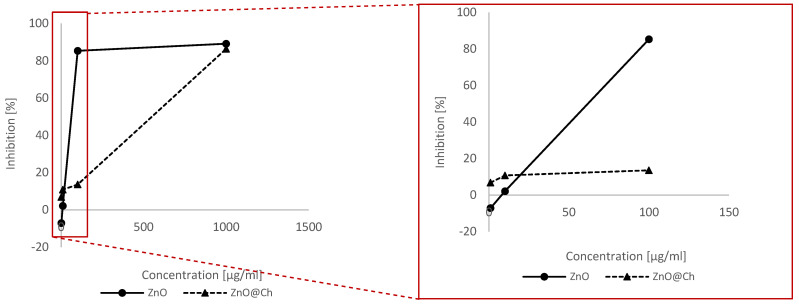
Depicts the trend of cytotoxicity values as a function of concentration, with a right magnification in the region from 0–100 μg/mL.

The cytotoxicity profile against 3T3 cells (mouse embryonic fibroblasts) of ZnO and ZnO@Ch is presented in Table 6 and Figure 9 as a percentage of proliferation inhibition.

The cytotoxicity trend is noteworthy because it demonstrates how coating leads to a decrease in cytotoxicity of up to the 100 μg/mL values. This effect can be attributed in part to the lower amount of zinc oxide in the adduct. However, the decrease in cytotoxicity is less than would be expected based on proportional reasoning. It is thus desirable that the full-fledged adduct also results in a decrease in inhibition due to the chemical nature of the newly synthesized adduct. Another possible explanation could lie in the increase in particle size, which decreases its uptake by cells.

### 2.8. Formulations

#### 2.8.1. Physicochemical Profile

The physicochemical characteristics of the emulsions containing ZnO and ZnO@Ch, respectively, were examined using the techniques described in the Materials and Methods section, and the results, in terms of pH, centrifuge stability, density, and viscosity of the emulsions, are summarized in Table 7. The emulsions obtained did not show substantial differences, except for a slightly higher pH for the emulsions containing ZnO, which was expected, given the amphoteric characteristics of ZnO.

#### 2.8.2. Filtering and Photocatalytic Activity

**Figure 10:**
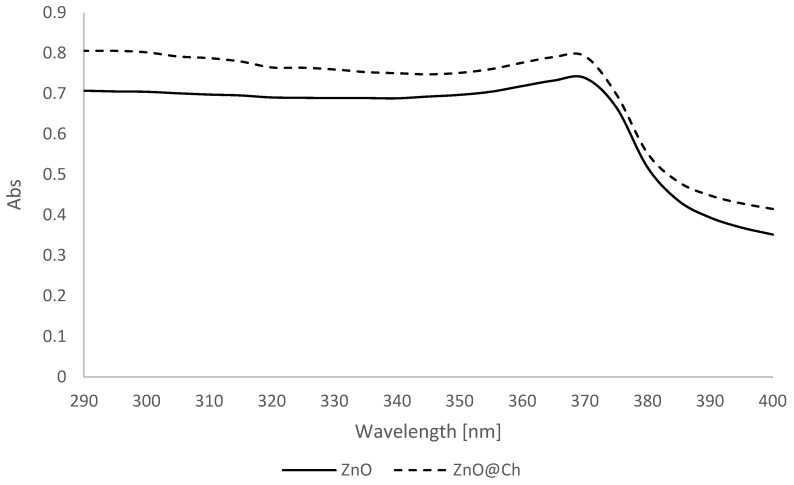
Absorbance for coated and uncoated ZnO.

**Figure 11:**
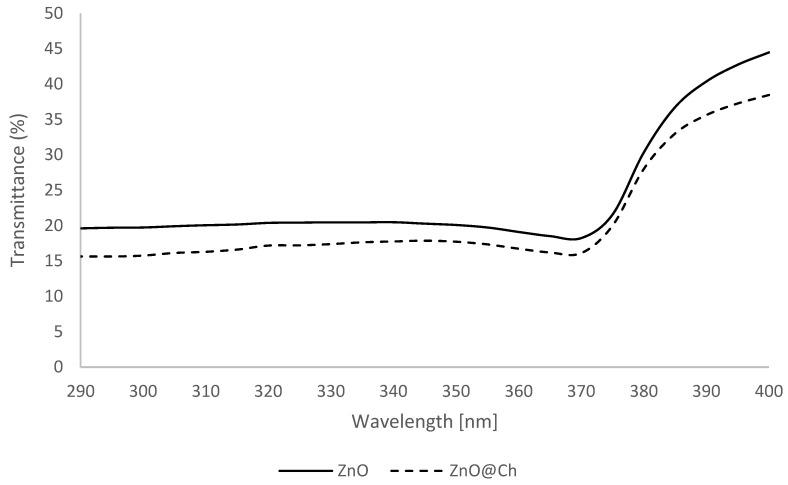
Transmittance for coated and uncoated ZnO.

**Figure 12:**
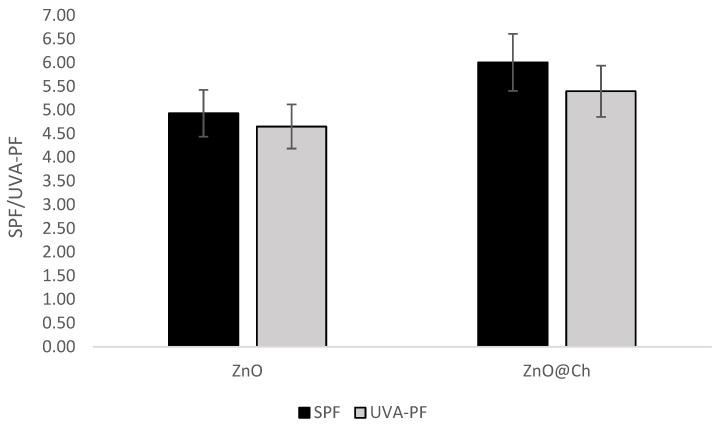
SPF and UVA-PF values for coated and uncoated ZnO. The increasing of SPF is statistically significant (*p* < 0.05).

The SPF evaluation demonstrated a statistically significant difference in the absorption values. Please refer to Figure 10 and Figure 11 for a side-by-side analysis of the absorption and transmittance of the two emulsions. Figure 12 presents the SPF values of ZnO (SPF 4.93, UVA-PF 4.65, λc = 380 nm) and ZnO@Ch (SPF 6.01, UVA-PF 5.40, λc= 380 nm). The graph reveals a substantial increase in both SPF and UVA-PF values, but the resulting emulsion had a limited protective effect against solar radiation, and it did not meet the minimum requirements to be classified as ‘protective and effective’ according to the Cosmetics Europe recommendation (SPF value of at least 6, UVA-PF of at least 1/3 of the SPF). Although the objective of this study was not to create a marketable product, the synthesized adduct’s potential to enhance sunscreen efficacy at the same weight was explored.

## 3. Discussion

The primary objective of the study was to synthesize and characterize the ZnO@Ch adduct, focusing on its functionalization, stability, and potential applications in cosmetics, particularly as a sunscreen agent. On the other hand, the FTIR analysis already confirmed the successful functionalization of ZnO with chitosan, indicating a significant chemical interaction between the two components. This information is crucial for understanding the properties of the ZnO@Ch adduct. The decision to not perform thermal analysis on the isolated compounds and physical mixtures was justified, given the study’s focus on the ZnO@Ch adduct and its functionalization. The existing knowledge of the thermal properties of the individual components, coupled with comprehensive thermal analysis of the adduct, provided sufficient information to support the study’s objectives. This approach avoided redundancy and ensured that the research efforts were directed towards the most relevant and impactful aspects of the study. Functional photostability testing, which is also important to understand the behavior of our material, was also postponed to the next phase of the study, in vivo testing, based on the robust preliminary data obtained from various critical parameters, including photocatalytic activity, SPF, and UVA-PF measurements, alongside a comprehensive suite of other characterizations. These data provided compelling evidence that the formulation exhibited enhanced stability and efficacy under typical conditions of use. The rationale is further reinforced by the well-established photoprotective properties of the active ingredients, chitosan and zinc oxide (ZnO). Zinc oxide is widely recognized for its ability to provide broad-spectrum UV protection, with documented stability under UV exposure. Similarly, as stated above, chitosan has been explored for its film-forming and stabilizing properties in cosmetic formulations, which contribute to enhanced longevity of the product upon application. Photostability assessments will be more appropriate in subsequent phases, where they can further validate the product’s long-term performance under extended UV exposure conditions.

Thus, the ZnO@Ch conjugate was synthesized using ionotropic gelation, which involved combining ZnO with chitosan and TPP via sonication to ensure homogeneity and prevent aggregation. This method led to stable conjugates with desirable properties for cosmetic use. FTIR analysis confirmed the functionalization of ZnO with chitosan, while TGA, DSC, and Zeta potential analysis validated the chemical interaction between the two components. The conjugate exhibited improved stability at neutral and slightly alkaline pH values, and its particle size distribution was characterized using DLS, SEM, and ELS. The stability of the ZnO@Ch adduct in the dispersion was assessed using a multisample analytical centrifuge. The study revealed that the ZnO@Ch adduct displayed improved dispersion stability compared to pure ZnO, which is vital for its application in cosmetic formulations. The SPF of the adduct was measured using an in vitro method, revealing a higher SPF than pure ZnO, possibly due to synergistic effects between chitosan and ZnO. This suggests that the ZnO@Ch adduct can offer enhanced UV protection at a lower concentration of ZnO, providing both economic and environmental benefits. The adduct’s photocatalytic activity was evaluated by its ability to degrade Acid Blue 9 dye, showing reduced activity compared to pure ZnO. The decrease in this context is advantageous, as it suggests a reduced generation of reactive oxygen species, thereby enhancing the safety profile of the adduct for topical applications. The cytotoxicity of the adduct was evaluated by using the Neutral Red Assay in 3T3 cells, revealing significantly lower toxicity than pure ZnO, especially at high concentrations. This decrease in toxicity can be ascribed to the adduct’s reduced solubility and Zn2+ release, making it a more suitable option for use in cosmetics. Furthermore, the synthesized particles were initially investigated through an in vitro study and compared based on efficacy by evaluating two emulsions containing 2 wt% ZnO and ZnO@Ch. The comparison was carried out by using total weight rather than weight in relation to the amount of ZnO to determine if the new adduct could allow for the use of less ZnO, with undeniable economic and environmental benefits, and to minimize the dispersion of the product into the environment. The results obtained were promising, leading to SPF values that are significantly higher than those of simple ZnO. The observed increases can probably be attributed to synergistic effects, due at least in part to the change in the refractive index, caused by the coating and the contribution of chitosan to SPF activity. The study’s safety perspective demonstrated a clear decrease in photocatalysis, with dye retention of more than 99%, and a significant reduction in cytotoxicity, down to concentrations of 100 μg/mL. These exceptional values emphasize the adduct’s improved safety properties.

Finally, the uncoated ZnO nanoparticles (naked ZnO) present ecotoxicity concerns, especially in aquatic environments. As mentioned in the review by Chatzigianni et al. [13], these nanoparticles can release Zn^2+^ ions, which are responsible for the observed toxic effects on marine life, including coral reefs and certain algal species. Specifically, the toxic impact on corals arises from the reduction in zooxanthellae, the symbiotic algae that corals depend on for energy. This process mirrors the effects seen with organic UV filters, which similarly disrupt the symbiotic relationship and contribute to coral bleaching.

Moreover, ZnO nanoparticles, when exposed to UVA and UVB radiation, exhibit a photocatalytic effect that can generate reactive oxygen species (ROS). These ROS can damage cellular structures, including membranes, mitochondria, and DNA, in various aquatic organisms, particularly in species like Pseudokirchneriella subcapitata and *Chlorella* sp., which have shown increased sensitivity to Zn^2+^ ions.

In cosmetic formulations, addressing these ecotoxicological risks involves focusing on coating or encapsulating ZnO nanoparticles to reduce ion release and photocatalytic activity. This approach not only enhances the safety profile of ZnO in sun care products, but also aligns with environmental regulations, which aim to mitigate the impact of cosmetic ingredients on marine ecosystems.

This formulation may offer both enhanced UV protection and reduced environmental risk. However, future studies are necessary to verify these claims, especially in regard to the potential (eco-)toxicological effects of ZnO nanoparticles and the environmental safety of the presented formulation.

## 4. Materials and Methods

### 4.1. Materials

Klasse A glassware and laboratory supplies are as follows: spatulas, pipettes, beakers, and cylinders; Stove (Heraeus, Milan, Italy); Leukofix (BSN medical S.r.l., Agrate Brianza, Italy); UV-Vis V-730 (JASCO International Co., Ltd., Tokyo, Japan); Perkin Elmer FTIR Spectrum 100 (Perkin Elmer, Milan, Italy) equipped with ATR using a ZnSe Diamond (FTIR-ATR); HPLC LC-4000 (JASCO International Co., Ltd., Tokyo, Japan); H2O HPLC grade (Merk, Darmstadt, Germany), CH3CN HPLC grade (Merk, Darmstadt, Germany); EtOH HPLC grade (Merk, Darmstadt, Germany); Netzsch 409/C TGA-DSC (Erich NETZSCH GmbH & Co., Holding KG, Selb, Germany); Nicomp ZLS Z3000 DLS-ELS (Billerica, MA, USA); LUMiSizer^®^ multisample analytical centrifuge (L.U.M. GmbH, Berlin, Germany); Zeiss Sigma VP (EVO40) EDS (Inca Energy 300, Oxford Instruments, High Wycombe, UK) and Gemini (SEM460) Field Emission SEM (Oberkochen, Germany); PalosL120CG2 120Kv with thermoionic source of lanthanum hexafluoride (Thermofisher Intruments, Eindoven, The Netherland); Seven Compact pH meter (Mettler Toledo, Columbus, OH, USA); Densimeter Easy D30 (Mettler Toledo, Columbus, OH, USA); Silverson L2R turbo-emulsifier (Silverson Machines Ltd. Waterside, Chesham, Bucks, UK); Centrifuge RE.MI XS R-8D (REMI Group, Mumbai, India); Brookfield Viscometer DV2T (Brookfield, Middleboro, MA, USA); DU-65 Digital ultrasonic cleaner (Argolab, Carpi, Italy); Siringe Pump 74900 Series (Antylia Scientific, Vernon Hills, IL, USA); Ultrasonic homogenizer (Hielscher Ultrasonics GmbH, Teltow, Gemany); Demineralized water; Ethanol (Silcompa S.p.A., Correggio, Italy); Acetone (Merk KGaA, Darmstadt, Germany); Blue Acid 9 (Farmalabor S.r.l., Assago, Italy); ZnO (Eclipse^®^ Z1, Uviva Technologies, INCI: Zinc Oxide, Duisburg, Germany); CH_3_COOH (Merk KGaA, Darmstadt, Germany); TPP (Merk, Darmstadt, Germany); Brij 721 (INCI: Steareth-21, Croda International plc, Snaith, England, UK); Mulsifan CSA2 (INCI: Steareth-2, Zschimmer & Schwarz, Lahnstein, Germany); Cutina GMS (INCI: Glyceryl Mono Stearate, BASF SE, Ludwigshafen am Rhein, Germany); Cetearyl alcohol (INCI: Cetearyl alcohol, ACEF, Fiorenzuola d’Arda, Italy); Cetiol C5 (INCI: Coco-Caprylate BASF SE, Ludwigshafen am Rhein, Germany); D-Phantenol (INCI: Panthenol Milano Colori S.r.l., Cusano Milanino, Italy); Xanthan Gum FNCSP-PC (INCI: Xanthan Gum, Jungbunzlauer Suisse AG, Basel, Suisse); Isocide BAS (INCI: Benzyl alcohol, Dehydroacetic acid, Lehvoss Italia S.r.l., Origgio, Italy); Chitosan (INCI: Chitosan, Galeno S.r.l., Comeana, Italy); and Sodium hydroxide (ACEF, Fiorenzuola d’Arda, Italy).

### 4.2. Functionalization Process

In a three-necked flask equipped with a bubble condenser, 30 mL of water and 0.30 g of ZnO dispersed through ultrasonication were placed. Chitosan, dissolved in 20 mL of water and with a gradual addition of 99% CH_3_COOH under mechanical agitation, was added to a separate container. The pH of the solution was then checked and adjusted to maintain a level of around 5.5 using NaOH, ensuring that the resulting solution was clear.

The following steps are taken after adding the chitosan solution to the ZnO solution and homogenizing it using an ultrasonicator bath: preparing a pentasodium triphosphate (TPP) solution with a weight ratio of 1:1:2 for the components, and injecting it into the reaction mixture using a syringe pump at a flow rate of 1.5 mL/min. The solution is then sonified for another 30 min to ensure complete mixing and reaction. The reaction mixture is then divided into centrifuge tubes and subjected to a centrifuge cycle at 5000 rpm for 10 min to remove any excess supernatant. The recovered precipitate is then transferred to a crystallizer and dried in an oven at 80 °C to facilitate solvent evaporation. To further reduce the size of the polymer obtained, it is placed in a 20 mL flask, brought to volume with distilled water, and sonified for about 24 h. The sonication process promotes chemical reactions by shaking the powder so that it becomes denser and more uniform, and the resulting product is then subjected to further processing as required.

### 4.3. Characterization of the Adduct

Upon the development of the Zn adduct and prior to its incorporation into the cosmetic formulation, a range of tests and analyses were conducted to characterize the adduct and determine its key properties as a solar filter and booster.

#### 4.3.1. Fourier Transform Infrared Spectroscopy (FTIR)

The examination was carried out on Fourier-transform infrared spectrometer system equipped with ATR, through the direct loading of a test sample onto the ATR holder of the instrument. The scanning process took place between 3000 and 500 cm^−1^.

#### 4.3.2. Thermogravimetric Analysis (TGA) and Differential Scanning Calorimetry (DSC)

Thermogravimetric Analysis (TGA) is an analytical technique that enables the quantification of weight loss from a sample at a specific temperature. In the context of the polymer under investigation, TGA allows for the verification of the actual weight percentage of ZnO that is present in the synthesized polymer by combusting the chitosan.

DSC analysis provides valuable insights about the materials utilized by subjecting them to heating and recording the resulting values. In the case of the ZnO@Ch polymer, the graph revealed the presence of two distinct cross-links. Thermogravimetric Analysis (TGA) and Differential Scanning Calorimetry (DSC) were simultaneously performed on a Netzsch 409/C instrument The heating program was set to increase from 30 °C–1000 °C at a rate of 5 °C per minute. Samples, weighing approximately 15 mg, were placed in a rhodium crucible, and alumina was used for internal calibration. The measurements were carried out by using an air/N_2_ mixture (40/80 mL/min).

#### 4.3.3. Particle Size and Potential Z (DLS-ELS)

The hydrodynamic diameter and Z-potential (Z-pot) were evaluated using a multi-angle Nicomp ZLS Z3000). After drying the powders, a stock solution for each material was prepared by dispersing the powders in ultra-pure water at the final concentration of 200 mg/L, using a probe sonicator (UP-200S Hielscher Ultrasonics GmbH, Germany) in an ice bath at 200 W for 10 min (in 80% pulsed mode). To study the colloidal stability, the particle size analysis and Z-pot analysis have been conducted at five pHs (3, 5, 7, 9, 12), adding HCl (1 M) or NaOH (1 M) to adjust the pH according to the experiment. The pH was controlled after the addition of the particles with a pHmeter (Hanna Instrument HI5222, calibrated using standard at pH 4, 7, 10), and adjusted if needed with small amount of NaOH or HCl. For the Z-pot measurements, NaCl has been used as an electrolyte (10 mM), and the pHs of the solution were adjusted with HCl (1 M) or NaOH (1 M), as needed. The prepared samples are then bath-sonicated (Falc Instruments LBS1-H3, Bergamo, Italy) for 5 min and directly analyzed. For the particle size analysis, the scattered light was collected using an optical fiber set at a scattering angle of 90° (W = 25 mW and λ = 639 nm) for a minimum of 6 min at room temperature. The Z-pot characterization was conducted using the same instruments, in which we transferred the samples in a quartz cuvette (10 mm) and used a platinum electrode for the analysis, applying a 5 V electric field. The data have been elaborated by the Nicomp software using the Smoluchowski approximation.

#### 4.3.4. Sedimentation Rate

The stability of the dispersed system was assessed by employing the LUMiSizer^®^ multisample analytical centrifuge (L.U.M. GmbH, Berlin, Germany), which is coupled with an NIR spectrometer and utilizes STEP™ (Space and Time-resolved Extinction Profiles) technology. By tracking the transmission change in any part of the sample or monitoring the movement of any phase boundary, the behavior of individual samples can be compared and analyzed in detail. The experiments were conducted by preparing a stock solution of each formulation at the concentration of 10 mg/L in a solution at pH 7 with 10 mM of NaCl. Each solution has been dispersed using a probe sonicator in an ice bath under 200 W for 10 min (in 80% pulsed mode), and after 10 min the samples has been transferred in triplicate in a polypropylene cuvette. The measurement was performed at a speed of 2000 rpm for 100 min, the temperature set to 24 °C, the laser wavelength was set at 470 nm, and the spectra were acquired every 10 s, 600 times. The data acquired has been elaborated with the proprietary software SEPView (L.U.M. GmbH, Berlin, Germany).

#### 4.3.5. Surface Characterization: SEM-EDS and TEM

Chemical composition and surface particle morphologies were investigated using scanning electron microscopy (SEM) coupled with a detector for energy-dispersive X-ray spectroscopy (EDS). The samples were examined, operating at an acceleration voltage of 15–20 kV. To improve the surface conductivity of samples, and, consequently, image resolutions, the gold sputtering deposition treatment was applied through vacuum evaporation. The images were collected at a high vacuum and with a high electron beam voltage to achieve a maximum resolution. EDS system analysis was used to verify the presence of organic coating through elemental analysis/composition in a “spot mode”, in which the beam was localized on a single area manually chosen within the field of view.

#### 4.3.6. Emulsion Formulation

The development of a cosmetic emulsion formulation necessitated the creation and preparation of a base emulsion, which was executed in the following manner:

1. In a beaker, the oil phase components of phase A were weighed and heated to approximately 65 °C on a hot plate while being stirred, to facilitate dispersion and melt the ingredients.
2. The aqueous phase components of phase B were separately weighed and placed on a heating plate, where they were heated to between 70–75 °C.
3. The two phases were combined by pouring phase A into phase B under the mechanical stirring of a turboemulsifier at the desired temperature.
4. Stirring was continued until a homogeneous product was obtained, and then the mixture was cooled using a water bath.
5. The D phase component was added to the mixture at a temperature below 40 °C and stirred until fully incorporated.
6. Finally, the pH was adjusted as necessary.

Next, the emulsion was divided into two aliquots, to which 2.00% zinc oxide nano and 2.00% ZnO@Ch were added, respectively (Table 8).

### 4.4. Evaluation of the Formulation

Upon finalization of the preparation process, both formulations were assessed in terms of their quality parameters.

#### 4.4.1. pH Determination

A bench pH meter, calibrated using two or three standard buffer solutions (4.01, 7.00, and 11.00), was employed for pH measurement. It is essential to store the glass electrode immersed in a 3 M KCl solution to prevent dehydration of the membrane. The use of distilled water should be avoided, as it may extract hydrogen ions from within the electrode through osmosis.

#### 4.4.2. Determination of Viscosity

The viscosity of the formulated emulsions was determined using a Brookfield rotary viscometer equipped with an SC4-29 spindle This instrument measures the resistance of the fluid to flow, which is expressed by viscosity. Specifically, when the rotating element is set in motion, due to the viscosity of the fluid, a force torque is generated that exerts its pressure on the cylindrical vessel. The resulting torque depends on the rotational speed, the geometry of the impeller, and the viscosity of the sample. From the measurement of the intensity of the torque, the viscosity of the fluid can be accurately traced. The unit used to measure the dynamic viscosity of a fluid is the centipoise (cPs), which is equivalent to one-hundredth part poise or millipascal per second (mPa×s).

#### 4.4.3. Calculation of Density

Density is a measure of the mass (expressed in kilograms) contained within a volume of one cubic meter of substance, and it is determined using a digital densimeter. This is accomplished by filling the instrument with the substance to be analyzed via syringe. The analysis is then carried out automatically, with an accuracy of ±0.0005 g per cubic centimeter.

#### 4.4.4. Centrifugation Test

Emulsions, whether oil-in-water (O/A) or water-in-oil (A/O), are inherently unstable, with the force of gravity causing the small, dispersed droplets to aggregate and sediment, either at the bottom or at the surface. Over time, these processes, which include flocculation, coalescence, and sedimentation, can lead to the complete separation of the emulsion’s components into their original phases, resulting in a loss of product efficacy. Therefore, it is essential to conduct accelerated stability tests using a laboratory centrifuge. This instrument simulates the gravity that would be exerted on the compound over the course of a year, allowing for the observation of any separation and sedimentation phenomena between bodies of different densities. This application of Archimedes’ principle and Stokes’ law is crucial for ensuring the stability and quality of the emulsion. To perform the test, approximately 30 g were taken from each emulsion and placed in special Falcon™ tubes (Merck KGaA, Darmstadt, Germany). The tubes were then subjected to centrifugation for 10 min at 6000 RPM.

### 4.5. Determination of SPF Value

Determining the SPF value in vitro requires the use of Leukofix BSN medical tape, a transparent tape that allows UV-VIS light to pass through and simulates the human epidermis. The Diffey–Robson method is used to determine the SPF factor, which involves cutting a rectangle and creating a 5×5 cm-sized square [22]. The sides of the square are then demarcated, and 0.070g of formulated emulsion is applied to the tape and distributed evenly with a fingertip. The amount of emulsion distributed is greater than the required amount, but it is considered that a loss of product occurs during the distribution step on the sample. The next step is to determine the baseline, which is obtained by inserting two rectangles of Leukofix BSN medical plaster into the appropriate section so that they are crossed by both the reference radius and the sampling radius. One then proceeds with the actual analysis, which involves inserting the sample into the instrument and taking at least ten acquisitions, varying the position of the patch relative to the incident radiation from time to time. This is done to check whether the emulsion has been homogeneously distributed over the sample. For each formulation tested, at least three media are prepared, and all the analyses performed are repeated to ensure the reproducibility of the data obtained. The curves obtained are analyzed using the Spectra Analysis program (Jasco Europe), and the various spectra obtained in the UVA and UVB 290–400 nm range are superimposed. Of the ten measurements obtained, six are chosen, and the obtained average is calculated. The average spectral curve is then imported directly into the SPF calculation program for analysis.

These analyses were carried out in duplicate, both for the emulsion containing the ZnO@Ch polymer and for that containing only ZnO.

The determination of SPF is obtained by processing the data with Microsoft Excel, and the UVA/UVB ratio becomes an important parameter of analysis, which follows the Diffey–Robson equation and transforms the absorbance values within the range of 400–290 nm into an average absorbance curve with an SPF value.

The formula used is as follows (Equation (1)):

(1) $$SPF=\frac{\sum_{290}^{400}E\;(\lambda )\epsilon (\lambda )}{\sum_{290}^{400}E\left(\lambda \right)\epsilon \left(\lambda \right)T(\lambda )}$$

*E*(λ) refers to the spectral irradiance of sunlight measured at noon on a midsummer day at a latitude of 40 degrees, and ϵ(λ) denotes the action spectrum for erythema caused by sunlight obtained from Diffrey and McKinlay. Additionally, *T*(λ) represents the transmittance. Analysis of a product using this method yields the following three primary values: the ratio of UVA to UVB, the Sun Protection Factor (SPF), and the UVA Protection Factor. However, it should be noted that the in vitro test cannot replace the in vivo method, but it can be useful as a screening tool for selecting products.

### 4.6. Determination of Photocatalytic Activity

Regarding the determination of photocatalytic activity, the photodegradation trend of Acid Blue 9 dye or Bright Blue was monitored following exposure to photocatalyzed light from the adducts obtained using the previously described method [23]. Specifically, the photocatalytic activity of ZnO was evaluated by preparing a 1 × 10^−5^ M (0.004 g) solution of Brilliant Blue FCF 85% in a 500 mL flask of water. For the investigation, 0.1 g of ZnO (Eclipse Z1) was weighed, dissolved in a beaker with 40 mL of Brilliant Blue FCF, and placed under mechanical stirring for a few minutes. The same procedure was followed for ZnO@Ch, the previously synthesized polymer.

**Figure 13:**
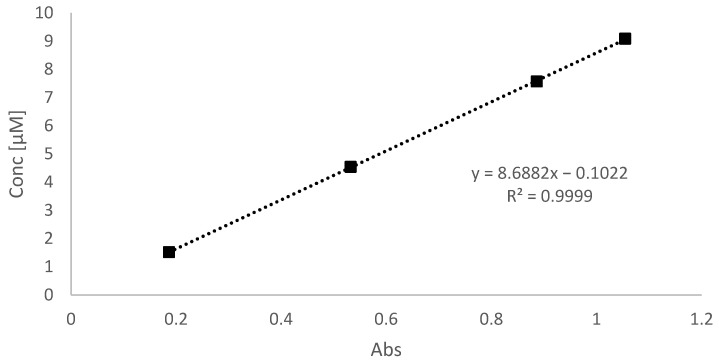
Calibration curve related to the AB9 concentration. The detections were obtained by considering the wavelength at 625 nm.

All samples were prepared in duplicate. One of the samples was wrapped in foil to test the adsorption of Bright Blue FCF by ZnO under dark conditions, while the other was subjected to UV light (366 + 254 nm lamp) to verify the photocatalytic activity. After 30 min of exposure to UVB radiation, about 25 mL of solution was taken and centrifuged at 6000 rpm for 10 min. The same procedure was repeated for the ZnO@Ch polymer. Subsequently, analysis was performed using the UV-Vis spectrophotometer. The spectra obtained were compared to each other, and the dye degradation was evaluated by measuring the absorbance value at 625 nm and considering the calibration curve above (Figure 13). The curve was made by considering the absorbance of AB9 at 3.0, 2.5, 1.5, and 0.5 µM.

The photocatalytic activity of each adduct was evaluated by performing the following analyses:

- Acid Blue 9 alone (Dark and UV-Vis): evaluation of the stability of the dye to light radiation.
- Zinc oxide alone (Dark and UV-Vis): evaluation of dye adsorption on the oxide surface.
- ZnO@Ch adduct (Dark and UV-Vis): evaluation of inhibition of photocatalytic activity.

The degradation percentages were calculated by considering the equation below (Equation (2)):

(2) $$\left(\%\right)=((A_{0}-A_{t})\times 100)/A_{0}$$

### 4.7. Evaluation of Cytotoxicity

The cytotoxicity of ZnO-based samples was assessed by using the Neutral Red Assay (NRU) on 3T3 cells (mouse embryonic fibroblasts). The NRU assay was carried out in DMEM (Dulbecco’s Modified Eagle Medium) containing 10% fetal calf serum (FCS), penicillin (100 U/mL), streptomycin (100 μg/mL), and glutamine (2 mM). The cells were seeded in triplicate in 96-well plates at a density of 7 × 10^3^ cells/well and treated for 48 h with various concentrations of the compounds (1 μg/mL, 10 μg/mL, 100 μg/mL, and 1000 μg/mL). The untreated cells served as a negative control and were considered to have 100% cell proliferation. After treatment, cells were washed with PBS and added to 250 µL of a solution of 25 µg/mL of NR. After 2 h, cells were washed again, and 150 µL of NRU-desorb solution (49% water, 50% ethanol 95%, and 1% glacial acetic acid) was added. The absorbance of the solution was then measured at 540 nm using a microplate reader, and values were converted to % of growth inhibition.

### 4.8. Statistical Analysis

The statistical analysis was conducted using a two-tailed Student’s t-test to compare the means between experimental groups. Each experiment was performed in triplicate to ensure reproducibility and statistical robustness.

The mean and standard deviation calculation are as follows: for each set of experimental replicates, the arithmetic mean ($\bar{X}$) was calculated using the following formula (Equation (3)):

(3) $$\bar{X}=\frac{1}{n}\sum_{i=1}^{n}X_{i}$$

where $X_{i}$ represents each individual measurement and *n*=3, corresponding to the number of replicates.

The standard deviation (SD) was calculated to quantify the dispersion of data points around the mean. The formula used is as follows (Equation (4)):

(4) $$SD=\sqrt{\frac{1}{n-1}\sum_{i=1}^{n}{\left(X_{i}-\bar{X}\right)}^{2}}$$

This measure reflects the variability within the triplicates.

The execution of statistical tests was as follows: all calculations, including mean, standard deviation, and the *t*-test, were performed using Microsoft Excel. The *t*-test was conducted using Excel’s built-in T.TEST function, with the assumption of unequal variances (heteroscedastic) where appropriate. Significance was defined at a *p*-value threshold of <0.05, and results were considered statistically significant when the *p*-value met this criterion.

## 5. Conclusions

This study aimed to investigate the consequences of applying a chitosan (a raw material side product deriving from food industry) coating on ZnO nanoparticles, an inorganic solar filter, in terms of its efficacy, safety, and sustainability. The initial phase of the research focused on creating a robust synthesis strategy and obtaining a coating through ionotropic gelation of chitosan with sodium triphosphate. Initial mechanical agitation was ineffective, leading to the use of in situ sonication to prevent large aggregates that interfere with emulsions. Once synthesized, the adduct was characterized, and its efficacy was evaluated using FTIR analysis for qualitative assessment and TGA/DSC to provide quantitative data. The results revealed that the ZnO@Ch species had an isoelectric point around neutrality, higher stabilization at pH 9, and a larger particle diameter, which explained the higher sedimentation rate. Safety tests showed that the ZnO@Ch adduct had lower photocatalytic and cytotoxic activities compared to ZnO, supporting its improved safety profile for skin applications. This preliminary work resulted in an adduct with a larger particle size and slower sedimentation rate than simple ZnO, yielding SPF values that are comparable, if not higher, than those of ZnO alone.

Finally, being a new raw material with unknown behavior in emulsion, in this first approach, it was decided to use 2% of the product to best evaluate the optimal methods of incorporation into the formula. Higher percentages at this stage of the research could compromise the final product, resulting in the loss of the entire synthesized adduct. On the other hand, in our experience, in vitro SPF measurement with inorganic filters performs better with lower concentrations, thus making it useful to increase booster effects [24]. In this regard, we are planning a formulation study after these preliminary results that requires conducting tests on volunteers. In this study, different concentrations of the new raw material will be investigated, as well as different formulations, to understand the performance of the same concentrations. Our tests showed that the ZnO@Ch adduct had lower photocatalytic and cytotoxic activities compared to ZnO, supporting its improved safety profile for skin applications. However, given the known concerns of the potential ecotoxicological impact of ZnO nanoparticles, further studies are required to fully assess the environmental safety of this new formulation.

## Figures and Tables

**Table 1:** Weight loss in TGA analysis.

Sample	Final Weight (%)	Water Loss (%)	Functionalization (%)
ZnO@Ch	55.78	8.27	44.22

**Table 2:** Z-potential in relation to pH.

	Z-Potential (mV)			
pH Value	ZnO		ZnO@Ch	
	Average	St. Dev.	Average	St. Dev.
3	−2.606	0.157	3.25	0.169
5	−1.54	0.125	2.08	0.147
7	25.69	1.077	−1.37	0.108
9	−2.612	0.188	−10.22	0.855
12	−6.007	0.205	−17.96	1.008

**Table 3:** Particle size in relation to different pH values.

	Particle Size (nm)			
pH Value	ZnO		ZnO@Ch	
	Average	St. Dev.	Average	St. Dev.
3	n.d.	n.d.	n.d.	n.d.
5	n.d.	n.d.	110	2
7	150	10	221.8	15.7
9	10.6	1.2	104.75	5.9
12	9.8	0.9	78.3	4.8

**Table 4:** Sedimentation rate for the coated and uncoated ZnO.

	Sedimentation Rate	
	µm/s	Dev. St.
**ZnO**	35.78	0.8
**ZnO@Ch**	60.62	3.5

**Table 5:** Acid Blue 9 degradation for the coated and uncoated ZnO.

Sample	Conc [M]	Abs (625 nm)	Degradation (%)	Dev. St. (%)
ZnO	4.57 × 10^−6^	0.548	54.30	5.8
ZnO@Ch	9.94 × 10^−6^	1.181	0.58	0.26

**Table 6:** Trend of cytotoxicity values as a function of concentration.

	3T3 Cells		
Sample	Concentration (µg/mL)	Inhibition (%)	Standard Deviation
Control	0	0	0
ZnO	1	−7.11	0.04
	10	2.12	0.01
	100	85.33	0.01
	1000	89.13	0.01
ZnO@Ch	1	6.82	0.14
	10	10.75	0.15
	100	13.55	0.11
	1000	86.39	0.02

**Table 7:** Chemical–physical parameters for the two different emulsions containing ZnO and ZnO@Ch.

Formulation	pH	Centrifuge (6000 rpm, 10 min)	Density (mg/mL)	Viscosity (cPs)
Base	5.89	Stable	0.982	32850
2% ZnO emulsion	6.72	Stable	0.989	33560
2% ZnO@Ch emulsion	6.59	Stable	0.991	32988

**Table 8:** Composition profiles of the two different formulations made with ZnO and ZnO@Ch.

Phase	Trade Name	INCI Name	Function	%
**A**	Brij 721	Steareth-21	Emulsifier	1.00–3.00
	Mulsifan CSA2	Steareth-2	Emulsifier	2.00–4.00
	Cutina GMS	Glyceryl Mono Stearate	Co-emulsifier	1.00–2.00
	Cetearyl alcohol	Cetearyl alcohol	Rheology modifier/co-emulsifier	4.00–6.00
	Cetiol C5	Coco-Caprylate	Emollient	19.00–21.00
**B**	Water	Aqua	Solvent	To 100
	D-Phantenol	Panthenol	Active substance	0.25–0.75
	Smoothflow	Xanthan Gum	Rheology modifier	0.10–0.50
**C**	Isocide BAS	Benzyl alcohol, Dehydroacetic acid	Preservative	0.50–2.00
**D**	ZnO/ZnO@Ch	Zinc Oxide/Zinc Oxide, Chitosan	Sunscreen filter	2.00
**E**	Sodium hydroxide 20%	Sodium Hydroxide, aqua	pH modifier	To pH 6.5

## References

[1] Shariatinia Z. (2019). Pharmaceutical applications of chitosan. Adv. Colloid Interface Sci..

[2] Bryuzgina E., Yartseva V., Bryuzgin E., Tuzhikov O., Navrotskiy A. (2023). Surface modification of film chitosan materials with aldehydes for wettability and biodegradation control. Polym. Bull..

[3] Kulka K., Sionkowska A. (2023). Chitosan based materials in cosmetic applications: A review. Molecules.

[4] Abd El-Hack M.E., El-Saadony M.T., Shafi M.E., Zabermawi N.M., Arif M., Elsaber Batiha G., Khafaga A.F., Abd El-Hakim Y.M., Al-Sagheer A.A. (2020). Antimicrobial and antioxidant properties of chitosan and its derivatives and their application: A review. Int. J. Biol. Macromol..

[5] Wei L., Tan W., Wang G., Li Q., Dong F., Guo Z. (2019). The antioxidant and antifungal activity of chitosan derivatives bearing Schiff bases and quaternary ammonium salts. Carbohydr. Polym..

[6] Han G., Mi Y., Ji X., Sun M., Tang H., Dong F., Guo Z. (2024). A novel chitosan antioxidant bearing sulfhydryl group: Synthesis, characterization and activity assessment. Int. J. Biol. Macromol..

[7] Ismailovi N., Tuba Kyan H., Ozturk A. (2024). A Novel Phytotherapy Application: Preparation, Characterization, Antioxidant Activities and Determination of Anti-inflammatory Effects by In vivo HET-CAM Assay of Chitosan-based DDSs Containing Endemic *Helichrysum pamphylicum* P.H. Davis & Kupicha Methanolic Extract. Curr. Drug Deliv..

[8] Huang Y.-L., Tsai Y.-H. (2020). Extraction of chitosan from squid pen waste by high hydrostatic pressure: Effects on physicochemical properties and antioxidant activities of chitosan. Int. J. Biol. Macromol..

[9] Zagloul H., Dhahri M., Bashal K., Khaleil M.M., Habeeb T.H., Khalil K.D. (2024). Multifunctional Ag_2_O/chitosan nanocomposites synthesized via sol-gel with enhanced antimicrobial, and antioxidant properties: A novel food packaging material. Int. J. Biol. Macromol..

[10] Battistin M., Dissette V., Bonetto A., Durini E., Manfredini S., Marcomini A., Casagrande E., Brunetta A., Ziosi P., Molesini S. (2020). A new approach to UV protection by direct surface functionalization of TiO_2_ with the antioxidant polyphenol dihydroxyphenyl benzimidazole carboxylic acid. Nanomaterials.

[11] de Araújo M.M., Schneid A.C., Oliveira M.S., Mussi S.V., de Freitas M.N., Carvalho F.C., Bernes Junior E.A., Faro R., Azevedo H. (2024). NLC-Based Sunscreen Formulations with Optimized Proportion of Encapsulated and Free Filters Exhibit Enhanced UVA and UVB Photoprotection. Pharmaceutics.

[12] Aranaz I., Alcántara A.R., Civera M.C., Arias C., Elorza B., Caballero A.H., Acosta N. (2021). Chitosan: An Overview of Its Properties and Applications. Polymers.

[13] Chatzigianni M., Pavlou P., Siamidi A., Vlachou M., Varvaresou A., Papageorgiou S. (2022). Environmental impacts due to the use of sunscreen products: A mini-review. Ecotoxicology.

[14] Levine A. (2021). Reducing the prevalence of chemical UV filters from sunscreen in aquatic environments: Regulatory, public awareness, and other considerations. Integr. Environ. Assess. Manag..

[15] Moeller M., Pawlowski S., Peterrsen-Thiery M., Miller I.B., Nietzer S., Heisel-Sure Y., Kellermannn M.Y., Schupp P.J. (2021). Challenges in Current Coral Reef Protection–Possible Impacts of UV Filters Used in Sunscreens, a Critical Review. Front. Mar. Sci..

[16] Yuan S., Huang J., Jiang X., Huang Y., Zhu X., Cai Z. (2022). Environmental Fate and Toxicity of Sunscreen-Derived Inorganic Ultraviolet Filters in Aquatic Environments: A Review. Nanomaterials.

[17] Sarbjot S., Rajneesh K., Brij B., Savita V., Samriti K. (2024). A Summarized Review of Formulation, in Vitro Evaluation of Sunscreen. IJISRT.

[18] Pasuch Gluzezak A.J., Dos Santos J.L., Stuchi Maria-Engler A., Rigo Gaspar L. (2024). Evaluation of the photoprotective and antioxidant potential of an avobenzone derivative. Front. Physiol..

[19] Buso P., Radice M., Baldisserotto A., Manfredini S., Vertuani S. (2017). Guidelines for the development of herbal-based sunscreen. Herbal Medicine.

[20] Bhattacharya S., Sherje A.P. (2020). Development of resveratrol and green tea sunscreen formulation for combined photoprotective and antioxidant properties. J. Drug. Deliv. Sci. Technol..

[21] Wazed A.S., Subbiyan R., Joshi M. (2011). Synthesis and characterization of chitosan and silver loaded chitosan nanoparticles for bioactive polyester. Carbohydr. Polym..

[22] Diffey B., Robson J. (1989). A New Substrate to Measure Sunscreen Protection Factors Throughout the Ultraviolet Spectrum. J. Soc. Cosmet. Chem..

[23] Battistin M., Pascalicchio P., Tabaro B., Hasa D., Bonetto A., Manfredini S., Baldisserotto A., Scarso A., Ziosi P., Brunetta A. (2022). A Safe-by-Design Approach to “Reef Safe” Sunscreens Based on ZnO and Organic UV Filters. Antioxidants.

[24] Manfredini S., Ziosi P., Vertuani S., Bustacchini S. (2009). A reflex technology to improve sun filter by SPF booster effect. J. Plast. Dermatol..

